# Precipitation Behavior of the Topologically Close-Packed Phase in the DD5 Superalloy during Long-Term Aging

**DOI:** 10.1155/2020/2569837

**Published:** 2020-03-06

**Authors:** Guiqun Liu, Xiaoli Zhang, Xinyi Wang, Yanxin Qiao

**Affiliations:** ^1^College of Material Science and Engineering, North Minzu University, Yinchuan 750021, China; ^2^School of Materials Science and Engineering, Jiangsu University of Science and Technology, Zhenjiang 212003, China

## Abstract

The precipitation behaviors of the topologically close-packed (TCP) phases in the bicrystal DD5 superalloy have been investigated. The results showed that the [001] crystallographic orientations are consistent with that of adjacent grains; however, the direction of the needle-like TCP phases is not consistent with that of the *γ* phase channels. The angle between needle-like TCP phases and *γ* phase channels is 45°, but the angle between the needle-like TCP phases of the adjacent grains is equal to the misorientation of the adjacent grains. Furthermore, during long-term aging, the needle-like TCP phases gradually decompose and transform into globular and short rod-like phases. The TCP phases precipitate preferentially in the dendrite. It is difficult to precipitate at the interdendrite/grain boundary, which is caused by the segregation of the constituent elements of the TCP phase to the dendrite.

## 1. Introduction

Superalloy is a kind of high-alloying iron-based, nickel-based, or cobalt-based metal material, which can withstand large complex stress above 600°C and has certain surface stability [1]. Nickel-based single-crystal superalloys have been widely used as jet engines and industrial gas turbine blades. In order to continuously improve its high temperature creep resistance, more and more refractory elements have been added to the superalloy. Presently, the known refractory elements such as Cr, Mo, W, and Re are good creep-strengthening elements. In particular, Re is recognized as the alloy element with outstanding strengthening effect. However, the addition of excessive refractory elements will significantly reduce the stability of the alloy structure.

The addition of excessive alloy elements such as Cr, Mo, W, and Re to the superalloy will cause the precipitation of intermetallic phases which have complex crystal structures rich in these refractory elements during long-term heat exposure or service. Because of their special dense structures, these phases are generally referred to as the topologically close-packed (TCP) phase [2]. The TCP phases of Ni-based single-crystal superalloys are *σ*, *μ*, P, and R [3, 4]. It is generally accepted that the TCP phase will deteriorate the creep properties of the alloy. So how to avoid the precipitation of the TCP phase is an important aspect of alloy design. Only by knowing more about the precipitation rules and characteristics of the TCP phase, we can avoid the precipitation of the TCP phase reasonably and effectively, so as to better optimize the design of the alloy.

Frank and Kasper [5, 6] first investigated the crystal structure characteristics of TCP phases and characterized the TCP phase by coordination polyhedron. These so-called Kasper polyhedrons have equilateral triangle surfaces and four atomic coordination numbers of 12, 14, 15, and 16, but in practice, polyhedron surfaces often deviate from equilateral triangles [2]. The structure of the TCP phase is composed of pentagonal or hexagonal antiprism arranged side by side. The antiprism can usually be regarded as some simple structural units, and using these structural units to characterize the structure of the TCP phase will greatly simplify the process of analysis [7–9].

In the past few decades, the precipitation behavior of the *μ* phase has been extensively studied [10–15]. Many structural defects have been found in the *μ* phase in some superalloys or intermetallic compounds, such as stacking faults, twin bands, microtwins, and second-phase symbiosis [11–13, 16–18]. However, there is little work on the detailed precipitation behavior of the P phase and R phase [3, 18–21].

In this paper, the TCP phase in the second-generation Ni-based superalloy DD5 is studied and the precipitation and evolution of the TCP phase are found and summarized. It provides evidence for further understanding the precipitation behavior and characteristics of the TCP phase in Ni-based single-crystal superalloys.

## 2. Materials and Methods

A second-generation single-crystal DD5 superalloy was used in this work. The chemical composition of the DD5 superalloy is listed in Table 1. The DD5 superalloy was produced by directional solidification and seeding so as to eliminate the effects of the grain boundary and crystallographic orientation on the TCP phase. In order to assure the directions of dendritic growth and thermal gradient to be accordant, the [001] direction of two seeds was aligned to the growth direction. The misorientation of the [010]/[100] direction of two seeds was about 20°. Since the [001] direction of the seed is parallel to the primary dendrites of the seed and the [010]/[100] direction of the seed is parallel to the secondary dendrites of the seed, the primary dendrites of two seeds are parallel to each other and parallel to the sample axis. The angle between the secondary dendrite arms of two seeds is about 20°. The dimension of the specimen was in size of 10 × 10 × 300 mm.

A Bridgman high-rate solidification (HRS) furnace [22, 23], modified with two hot zones (upper zone and lower zone), was used in this study. The ceramic model was mounted on the water-cooled copper chill plate. The temperatures of the upper zone and lower zone were 1480°C and 1580°C, respectively. The master alloy ingot was heated to 1580°C and kept at the temperature for 5 min. Subsequently, the molten alloy was poured into the preheated mold. To ensure the system to attain a thermal equilibrium, casting samples were directionally solidified after 10 min of the pouring and pulled down in a withdrawal speed of 6 mm/min. When the temperature of the casting samples dropped to room temperature, the ceramic shell was broken and the bicrystal DD5 samples were taken out.

To determine the location of the grain boundary of the bicrystal DD5, all samples were macroetched with a mixture of HCl and H_2_O_2_ (volume ratio 5 : 1). Subsequently, the misorientation of the bicrystal DD5 superalloy was examined by the Electron Back Scattered Diffraction (EBSD) technique in a Scanning Electron Microscope (SEM). After microetching in a mixture of 100 ml HCl, 100 ml H_2_O, 5 ml H_2_SO_4_, and 2 g CuSO_4_, the dendrite structure was observed using an optical microscope (OM). Finally, the TCP phase was observed by a SEM. The angle of *γ* (needle-like TCP) phases between two grains was measured by the analysis software of Image-Pro Plus.

To study the element segregation of as-cast DD5, the concentrations of each chemical element in dendrite and interdendrite were measured three times by the Electron Probe Micro Analyzer (EPMA). Then, the average value of the three measurements was taken. The segregation ratio was the average concentration of every element at dendrite divided by that at interdendrite.

The vacuum heat treatment of the bicrystal DD5 was as follows: 1310°C/2 h, air cooling; 1130°C/4 h, air cooling; and 900°C/16 h, air cooling. The long-term aging was 950°C for 100 h, 500 h, 1000 h, and 2000 h.

## 3. Results and Discussions

**Figure 1:**
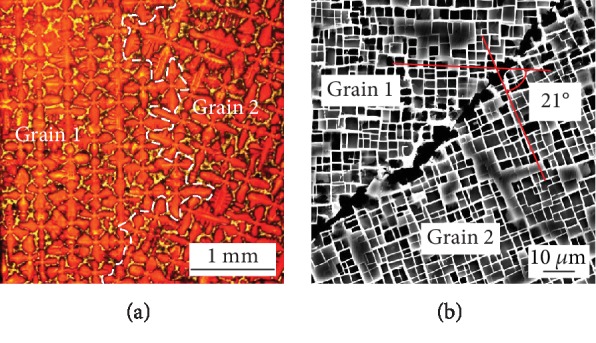
(a) Metallographic micrograph of as-cast bicrystal specimen and (b) SEM micrograph of the microstructure after standard heat treatment.

The cross-sectional microstructure of as-cast bicrystal DD5 is shown in Figure 1(a). It is the typical dendritic structure of the <001> direction in grains 1 and 2, and the “white phase” of the interdendritic region is *γ*/*γ*′ eutectic. The white dotted line between the two grains is the grain boundary. The angle between the secondary dendritic arms of both sides against the grain boundary is about 21°.

The cross-sectional microstructure of as-cast DD5 after vacuum heat treatment is shown in Figure 1(b). In grains 1 and 2, the white channel is the *γ* phase and the black cube phase is the *γ*′ phase. The *γ*/*γ*′ eutectic at the grain boundary has completely disappeared, and the irregular block-like black *γ*′ phases appear. It is worth noting that the direction of the white *γ* phase in each grain is consistent with that of the secondary dendrite arm in Figure 1(a), and the angle between the two grains is about 21°. In other words, under the condition that the [001] crystallographic orientation of the adjacent grains is the same, the angle between the phase channels of the adjacent grains is consistent with that between the secondary dendritic arms of the adjacent grains.

**Figure 2:**
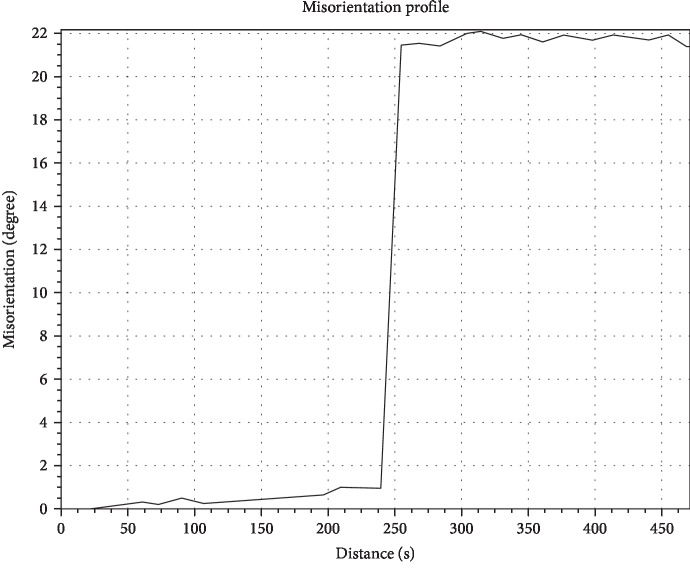
Misorientation of the as-cast bicrystal DD5 specimen measured by the line scanning of EBSD.

The precise misorientation of the two grains in Figure 1 is shown in Figure 2. The precise misorientation of the two grains is 21°. This is in good agreement with the conclusion obtained from Figure 1. When the [001] crystallographic orientation of the adjacent grains is the same, the misorientation of two adjacent grains is equal to the angle between the *γ* phase channels of two adjacent grains and the angle between the secondary dendritic arms of two adjacent grains.

**Figure 3:**
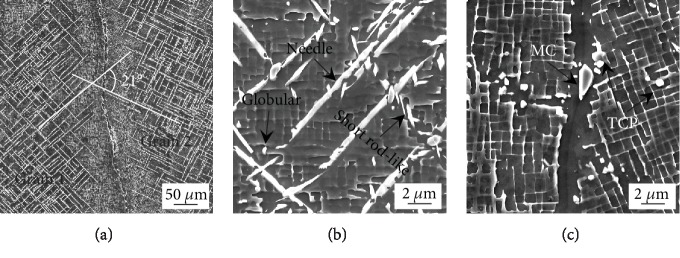
(a) SEM observation of the needle-like TCP phase in the bicrystal DD5 superalloy annealed at 950°C for 1000 h. (b) SEM micrograph of three TCP morphologies (needle-like, short rod-like, and globular). (c) SEM micrograph of the globular TCP phase and MC carbide at the grain boundary.

Figure 3 presents the morphologies of the TCP phase that precipitated in the bicrystal superalloy during long-term aging at 950°C for 1000 h. As is shown in Figure 3(a), there was almost no TCP phase at the grain boundary, and a large number of needle-like TCP phases are parallel or perpendicular to each other precipitate on the dendrite. The needle-like TCP phases precipitate along the <110> crystal direction on the {111} crystal plane, and they are parallel or perpendicular to each other on the {001} crystal plane. In addition, the angle between the needle-like TCP phases of two adjacent grains is about 21°. This is consistent with the results in Figure 2. However, the direction of the needle-like TCP phase is not consistent with that of the *γ* phase channel, but at an angle of 45°, as shown in Figure 3(b). The TCP phase is needle-like, short rod-like, and granular, as is shown in Figure 3(b). There are bigger MC carbides and small globular TCP phases at the grain boundary.

**Figure 4:**
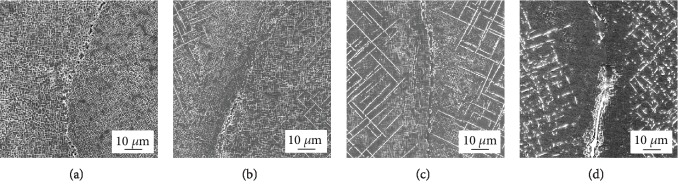
SEM micrographs of TCP phases in the grain boundaries annealed at 950°C for different hours: (a) 100 h, (b) 500 h, (c) 1000 h, and (d) 2000 h.

Figure 4 presents the morphologies of the TCP phase that precipitated near the grain boundaries during long-term aging at 950°C. After aging for 100 h, there is no any TCP phase that precipitated at the dendrites and grain boundary, and there is a large piece of the irregular *γ*′ phase at the grain boundary, as shown in Figure 4(a). After aging for 500 h, there is a small amount of needle-like TCP phases parallel or perpendicular to each other and small granular TCP at the dendrite, and there is no TCP phase that precipitated at the grain boundary, as shown in Figure 4(b). After aging for 1000 h, a large number of needle-like TCP phases perpendicular to each other and big granular TCP appear at the dendrite and massive carbides begin to precipitate on the grain boundaries, as shown in Figure 4(c). After aging for 2000 h, the needle-like TCP phase at the dendrite decomposes into granular and short rod-like phases, as shown in Figure 4(d).

**Figure 5:**
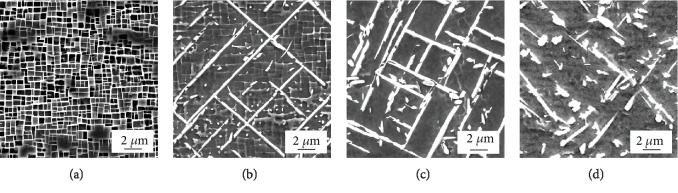
SEM micrographs of TCP phases in the dendrite trunks annealed at 950°C for different hours: (a) 100 h, (b) 500 h, (c) 1000 h, and (d) 2000 h.

Figure 5 shows the morphologies of the TCP phase that precipitated on the dendrite during long-term aging at 950°C. After aging for 100 h, there is no TCP phase that precipitated in the dendrite trunk, only the black *γ*′ phase and white *γ* phase, as shown in Figure 5(a), which is consistent with that of the low magnification of Figure 4(a). After aging for 500 h, needle-like, granular, and short rod-like TCP phases precipitate at the dendrite trunk as shown in Figure 5(b). However, granular and short rod-like TCP phases are too small to be seen clearly; only the needle-like TCP phase can be seen in Figure 4(b). After aging for 1000 h, the needle-like TCP phases begin to decompose and transform into granular and short rod-like ones, as shown in Figure 5(c). However, at low magnification (Figure 4(c)), granular and short rod-like TCP phases are too small to be seen clearly; only the needle-like TCP phase can be seen. After aging for 2000 h, as shown in Figure 5(d), almost all needle-like TCP phases decompose into granular and short rod-like ones, which is consistent with that of the low magnification of Figure 4(d).

It can be derived from Figures 4 and 5 that with increasing aging time, the TCP phase gets coarser. This phenomenon is similar to the previous investigations [24, 25]. The needle-like TCP phases first coarsened and then decomposed. The granular TCP coarsened first and then branched.

**Figure 6:**
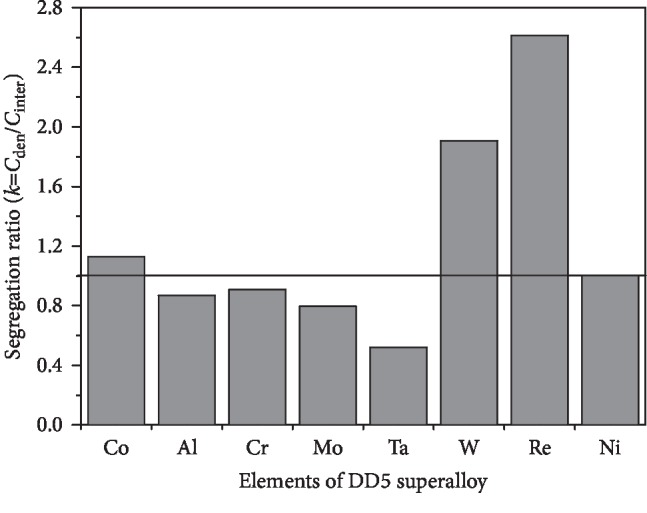
The element segregation of the as-cast DD5 superalloy.

It can be inferred from Figure 4 that TCP phases preferentially precipitate at the dendritic trunk, but it is difficult to precipitate at the interdendrite/grain boundary. This is caused by the segregation of the constituent elements of the TCP phase. The chemical composition of the TCP phase is made up of Cr, Mo, W, and Re. For superalloy containing Re, the TCP phase usually has a high content of Re. The element segregation of the as-cast DD5 superalloy is shown in Figure 6. The segregation ratio of Re and W is 2.6 and 1.9, respectively. The segregation ratio of Cr and Mo is 0.9 and 0.8, respectively, which is close to 1. The dendritic segregation of W and Re is serious, while the interdendritic segregation of Cr and Mo is not as intense as that for W and Re. In addition, the TCP phase contains more Re, so the TCP phase preferentially precipitates at the dendrite trunk.

## 4. Conclusions

1. When the [001] crystallographic orientation of two adjacent grains is the same, the misorientation of the adjacent grains is not only the angle between the *γ* phase channel of two adjacent grains but also the angle between the secondary dendrite arms of two adjacent grains. Although the direction of the needle-like TCP phase is not consistent with that of the *γ* phase channel, but at an angle of 45°, the angle of the needle-like TCP phase of two adjacent grains is equal to the misorientation of two adjacent grains
2. In the process of long-term aging, the needle-like TCP phase gradually decomposes and transforms into granular and short rod-like ones
3. The TCP phases precipitate preferentially at the dendrite, but it is difficult to precipitate at the interdendrite/grain boundary, which is caused by the segregation of the constituent elements of the TCP phase to the dendrite

## Figures and Tables

**Table 1:** The chemical compositions of the DD5 superalloy (wt%).

Cr	Co	Mo	W	Al	Ta	Re	Hf	C	Ni
7.15	7.8	1.5	5.05	6.3	6.6	3	0.15	0.022	Bal.

## Data Availability

The data used to support the findings of this study are available from the corresponding author upon request.
